# A phase II study of antiangiogenic therapy (Apatinib) plus chemotherapy as second‐line treatment in advanced small cell lung cancer

**DOI:** 10.1002/cam4.5217

**Published:** 2022-09-09

**Authors:** Yinghui Xu, Xu Wang, Chao Sun, Zhiru Gao, Hua He, Shi Qiu, Ye Guo, Xiaohui Ma, Junya Song, Kewei Ma

**Affiliations:** ^1^ Cancer Center The First Hospital of Jilin University Changchun China

**Keywords:** angiogenesis, apatinib, small cell lung cancer (SCLC), targeted therapy, VEGFR2

## Abstract

**Introduction:**

Currently, only a few options are available for the treatment of patients with small‐cell lung cancer (SCLC) after the failure of first‐line platinum‐based chemotherapy. The present study aimed to evaluate the efficacy and safety of apatinib plus chemotherapy for second‐line treatment of advanced SCLC.

**Patients and Methods:**

This prospective clinical trial recruited patients treated with apatinib plus second‐line chemotherapy until disease progression or intolerable toxicity. Logrank test power analysis was used for calculating samples. The primary endpoint was progression‐free survival (PFS), and the secondary endpoints were objective response rate (ORR), disease control rate (DCR), overall survival (OS), and safety.

**Results:**

A total of 29/31 enrolled patients were available for response evaluation until October 2019. The ORR and DCR were 27.59% (8/29) and 96.55% (28/29), respectively. The median PFS and OS were 7.36 months and 14.16 months, respectively, indicating better efficacy compared with the standard second‐line chemotherapies. The most common adverse events (AEs) were neutropenia (41.94%, 13/31), followed by leucopenia (35.48%, 11/31) and thrombocytopenia (25.81%, 8/31). The grade 3–4 AEs occurred in 12 (38.71%) patients, of which neutropenia (19.35%, 6/31), leucopenia (9.68%, 3/31), and proteinuria (6.45%, 2/31) were most common. Patients receiving an initial dose of apatinib 250 mg had a better tolerance.

**Conclusion:**

Antiangiogenic therapy plus chemotherapy had encouraging efficacy in advanced SCLC patients, which provides an insight into the current status of second‐line therapy in SCLC.

## Graphical abstract

Antiangiogenic therapy (Apatinib) plus chemotherapy had better efficacy compared with standard second‐line chemotherapies for advanced SCLC patients. No more intolerable toxicities were observed for this new regimen, which should provide a highlight view on the current status of second‐line therapy in SCLC.

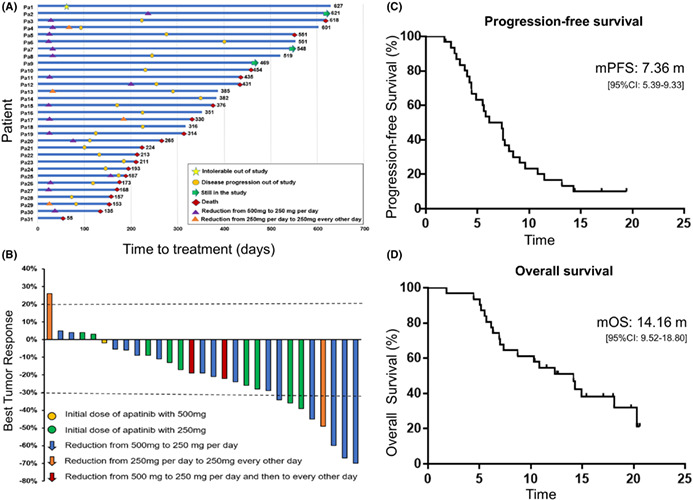

## 1 INTRODUCTION

Lung cancer regarded as one of the most common causes of cancer‐related mortality.^1^ Small cell lung cancer (SCLC) is a highly aggressive malignant disease that accounts for 15%–20% of lung cancer worldwide.^2^ Although SCLC is sensitive to initial chemotherapy, such as etoposide and platinum, the majority of these patients have a high probability of recurrence within 6 months after 4–6 cycles of first‐line chemotherapy and do not respond to subsequent chemotherapy. Consequently, such SCLC patients have a median overall survival (OS) of only 1 year.^3^ Therefore, new second‐line therapeutics are needed to improve the prognosis of SCLC. Angiogenesis is frequent in non‐small cell lung cancer (NSCLC) and is associated with aggressive disease.^4^ Reportedly, high vascular endothelial growth factor (VEGF) expression was observed in about 80% of SCLC tissues.^5^ Moreover, VEGF level is associated with microvessel density in cancer tissues and serves as an independent poor prognostic factor for SCLC patients.^6^ Therefore, inhibition of tumor angiogenesis is considered a new valuable approach for this disease. Bevacizumab is the first angiogenesis inhibitor approved for the treatment of NSCLC and has been tried for SCLC treatment. Previous studies showed that the addition of bevacizumab to chemotherapy for the treatment of SCLC could significantly improve the progression‐free survival (PFS), but has less benefit in OS.^7^, ^8^ Furthermore, some intravenous antiangiogenic agents, such as thalidomide, endostar, and pazopanib, may add toxicity and increase the risk of death, thus also limiting their development and prospective application.^9^, ^10^, ^11^

Apatinib is an oral tyrosine kinase inhibitor (TKI) that specifically inhibits the vascular endothelial growth factor receptor‐2 (VEGFR‐2). As a new antiangiogenic drug, apatinib has shown promising clinical efficacy for SCLC patients. Dai et al. used apatinib as the maintenance treatment following the first‐line chemotherapy in SCLC patients, which showed promising efficacy and safety.^12^ Hong et al. found that apatinib (500 mg once daily) was effective for pretreated extensive‐stage SCLC with PFS of 4.5 months in a retrospective study.^13^ Xu et al. discovered favorable efficacy (PFS 3.0 months, OS 5.8 months) of apatinib (500 mg once daily) and acceptable safety in previously treated SCLC patients in a prospective study.^14^ Regarding combination regimens, previous studies indicated that apatinib combined with chemotherapy showed potential antitumor activity in advanced NSCLC. Zhang et al. demonstrated that apatinib plus vinorelbine might be an effective regimen for patients with advanced NSCLC after the failure of second‐line therapy.^15^ Yang et al. reported that apatinib (250 mg/day) in combination with six cycles of pemetrexed‐platinum, followed by monotherapy with apatinib until progressive disease or intolerable toxicity, showed satisfactory efficacy and safety in advanced non‐squamous NSCLC (PFS 7.7 months, OS 20.1 months).^16^

However, the effect of apatinib plus chemotherapy in SCLC has not yet been defined. Apatinib might represent a new treatment for patients with SCLC, who have fewer options, especially in the event of relapse. In a retrospective analysis, the addition of apatinib to single‐agent chemotherapy showed a modest improvement in efficacy. Therefore, we designed a phase II, prospective, single‐center, single‐arm, clinical trial to study the clinical efficacy and toxicity of apatinib plus chemotherapy in pretreated patients with advanced SCLC.

## 2 PATIENTS AND METHODS

### 2.1 Patients

In this single‐arm, phase II prospective study, pretreated patients with advanced SCLC were selected. The eligibility criteria were as follows: (1) 18–75‐years‐old; (2) Eastern Cooperative Oncology Group (ECOG) performance score ≤2; (3) Pathological examination confirmed SCLC; (4) Limited stage patients could receive thoracic radiation, surgery, or any other curative intent therapy as first‐line therapy; (5) Disease progression after first‐line chemotherapy; Platinum‐sensitive patients (relapse ≥6 months after first‐line chemotherapy) or platinum‐resistant patients (relapse <6 months after first‐line chemotherapy); (6) Absence of brain metastasis or asymptomatic brain metastasis was allowed; (7) Normal function of major organs (appropriate hematological, coagulation, hepatic, and renal function). The key exclusion criteria were as follows: (1) Previous treatment with apatinib or other antiangiogenetic agents; (2) Patients with other malignant tumors; (3) Patients allergic or intolerant to the study drugs.

Generally, the first‐line therapy including surgery or radiotherapy refers to the initial treatment of patients with confirmed SCLC, we listed some conditions for the first‐line therapy including surgery or radiotherapy. (1) Some very early limited‐stage SCLC patients with clinical stage T1‐2N0M0 can undergo surgical treatment if the body can tolerate it, and then chemotherapy and radiotherapy should be further performed after the surgery^17^ (1 patient in our study received surgery as initial treatment); (2) Concurrent chemoradiotherapy or sequential chemoradiotherapy should be performed for the patients with limited‐stage SCLC who are not suitable for surgery (16 patients received sequential chemoradiotherapy as initial treatment); (3) Thoracic radiotherapy is also suitable for some extensive‐stage SCLC patients with residual lesion after chemotherapy to further reduce the risk of thoracic tumor recurrence and prolonging patient survival^18^, ^19^ (7 patients received chemotherapy as initial treatment). Recurrent disease progression or relapse after first‐line therapy could be included in our study.

This study was performed following the Declaration of Helsinki and the Principles of Good Clinical Practice. The protocol was reviewed and approved by the institutional review board of The First Hospital of Jilin University, Changchun, China and written informed consent was obtained from each participant. The ClinicalTrials.gov identifier was NCT03547804.

### 2.2 Treatment schedule

Initially, 18 patients were administered 500 mg apatinib once per day plus four cycles of chemotherapy, followed by single apatinib (500 mg) treatment until disease progression, death, intolerance, or consent withdrawal. Because of the intolerance to the dosage regimen at the primary stage, 13 patients were subsequently treated with 250 mg apatinib once daily plus four cycles of chemotherapy at the beginning of the treatment, followed by single apatinib (250 mg) treatment until disease progression, death, intolerance, or consent withdrawal. The dosage of apatinib was further adjusted to 250 mg every alternate day if a grade 3–4 adverse effect was observed. The chemotherapeutic agents were standard second‐line chemotherapy drugs, such as irinotecan (60 mg/m^2^, day 1 and 8 q3 weekly), docetaxel (75 mg/m^2^, q3 weekly), or others,^20^ selected as appropriate. The flow chart of the study design is shown in Figure S1.

### 2.3 Efficacy and toxicity evaluation

All the patients were followed up until disease progression or death. Tumor response was evaluated by imaging every two cycles of treatment (every 6 weeks) according to the Response Evaluation Criteria in Solid Tumors guidelines version 1.1 (RECIST1.1). Any toxicity was observed and graded according to the National Cancer Institute Common Toxicity Criteria Version 4.0 (NCI CTCAE 4.0). All the recorded data were evaluated and checked by our clinicians. Other assessments, including physical examination, measurement of vital signs, complete blood count, biochemistry, and routine urine analysis, were also performed after every two cycles of treatment.

### 2.4 Study endpoints

The primary endpoint of this study was PFS (the time from the start of treatment to the time of disease progression or death). The secondary endpoints included objective response rate (ORR) (proportion of patients achieving complete response or partial response), disease control rate (DCR) (proportion of patients with complete or partial response or stable disease), OS (the time from the start of treatment to the time of death, follow‐up failure, or follow‐up deadline), and safety.

### 2.5 Statistical analysis

Previous large‐scale clinical studies indicated that the longest PFS of second‐line single‐agent chemotherapy for SCLC lasted about 4 months (Table S1). We assumed that adding apatinib to standard second‐line chemotherapy would increase the PFS to about 7 months. With 30 patients who treated with apatinib plus chemotherapy, the study has approximately 80.58% power to detect an effect size of PFS = 7 months under the null hypothesis of PFS = 4 months, at a type I error (alpha) associated with this one‐sided test was 0.025. Thirty‐one patients were ultimately enrolled in our study. SPSS version 26.0 was used for data analysis. Kaplan–Meier method was used to analyze the survival trend. *p* < 0.05 indicated statistically significant difference. Logrank test power analysis was used for calculating samples.

## 3 RESULTS

### 3.1 Patient characteristics

A total of 31 patients were enrolled in this study from May 31, 2018 to October 25, 2019. The baseline characteristics of the subjects are listed in Table 1. The median age of the cohort was 61 (range, 47–74) years, and the majority were males (21/31, 67.74%). Among them, 13 patients had a smoking history (13/31, 41.94%), most had good performance status (ECOG 0–1) (29/31, 93.55%), 17 were diagnosed with limited‐stage SCLC (17/31, 54.84%), and 14 were diagnosed with extensive‐stage SCLC (14/31, 45.16%). For the initial treatment, 1 patient(3.23%)was treated with surgery and postoperative chemotherapy, 23 patients (74.19%) were treated with chemotherapy combined with sequential radiotherapy (among of them, 16 patients were diagnosed with limited stage, another seven patients were diagnosed with extensive‐stage), and the remaining seven patients (22.58%) with extensive‐stage were treated with chemotherapy alone. In addition, for specific chemotherapy regimen, 21 patients were treated with etoposide‐cisplatin, and 10 were treated with etoposide‐carboplatin chemotherapy regimen. In addition, most patients received 4–6 cycles of first‐line chemotherapy (30/31, 96.77%). Regarding the first‐line platinum‐treated relapse, 16 patients were platinum‐sensitive (16/31, 51.61%) and 15 were platinum‐resistant (15/31, 48.39%). Subsequently, 22/31 (70.97%) completed apatinib treatment combined with four cycles of chemotherapy, and the remaining 9/31 (29.03%) patients did not complete all treatments due to intolerable toxicity or disease progression.

**TABLE 1:** Clinical baseline characteristics of total patients (*n* = 31)

Characteristics	Number of patients (%)
Gender	
Male	21 (67.74)
Female	10 (32.26)
Age, years	
<60	13 (41.94)
≥60	18 (58.06)
Smoking history	
Former/Ever	13 (41.94)
Never smoker	18 (58.06)
ECOG scores	
0–1	29 (93.55)
2	2 (6.45)
Stage	
Limited	17 (54.84)
Extensive	14 (45.16)
First‐line chemotherapy regimen	
Etoposide‐cisplatin	21 (67.74)
Etoposide‐carboplatin	10 (32.26)
First‐line chemotherapy cycle	
<4	1 (3.23)
4–6	30 (96.77)
Relapse type of first‐line regimen	
Platimum‐sensitive	16 (51.61)
Platimum‐resistant	15 (48.39)
Initial dose	
500 mg	18 (58.06)
250 mg	13 (41.94)
Complete four cycles of chemotherapy	
Yes	22 (70.97)
No	9 (29.03)

Abbreviations: ECOG, Eastern Cooperative Oncology Group.

### 3.2 Treatment outcomes

**FIGURE 1:**
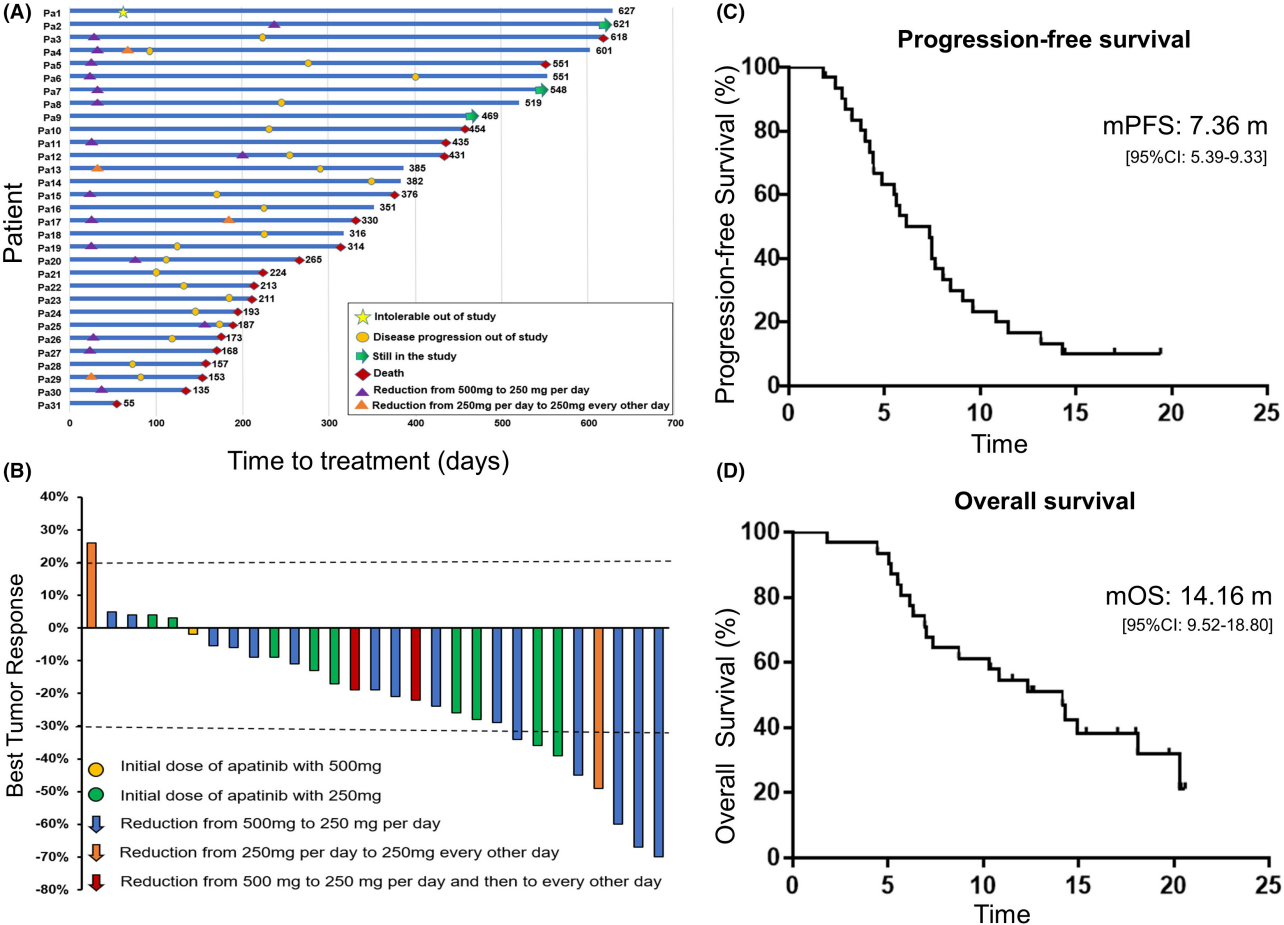
Clinical outcomes and survival analysis of the patients. (A) Swimmers plot of survival outcomes of 31 patients. (B) Waterfall plot of best response among the evaluable 29 patients. Kaplan–Meier plots of (C) PFS and (D) OS.

**FIGURE 2:**
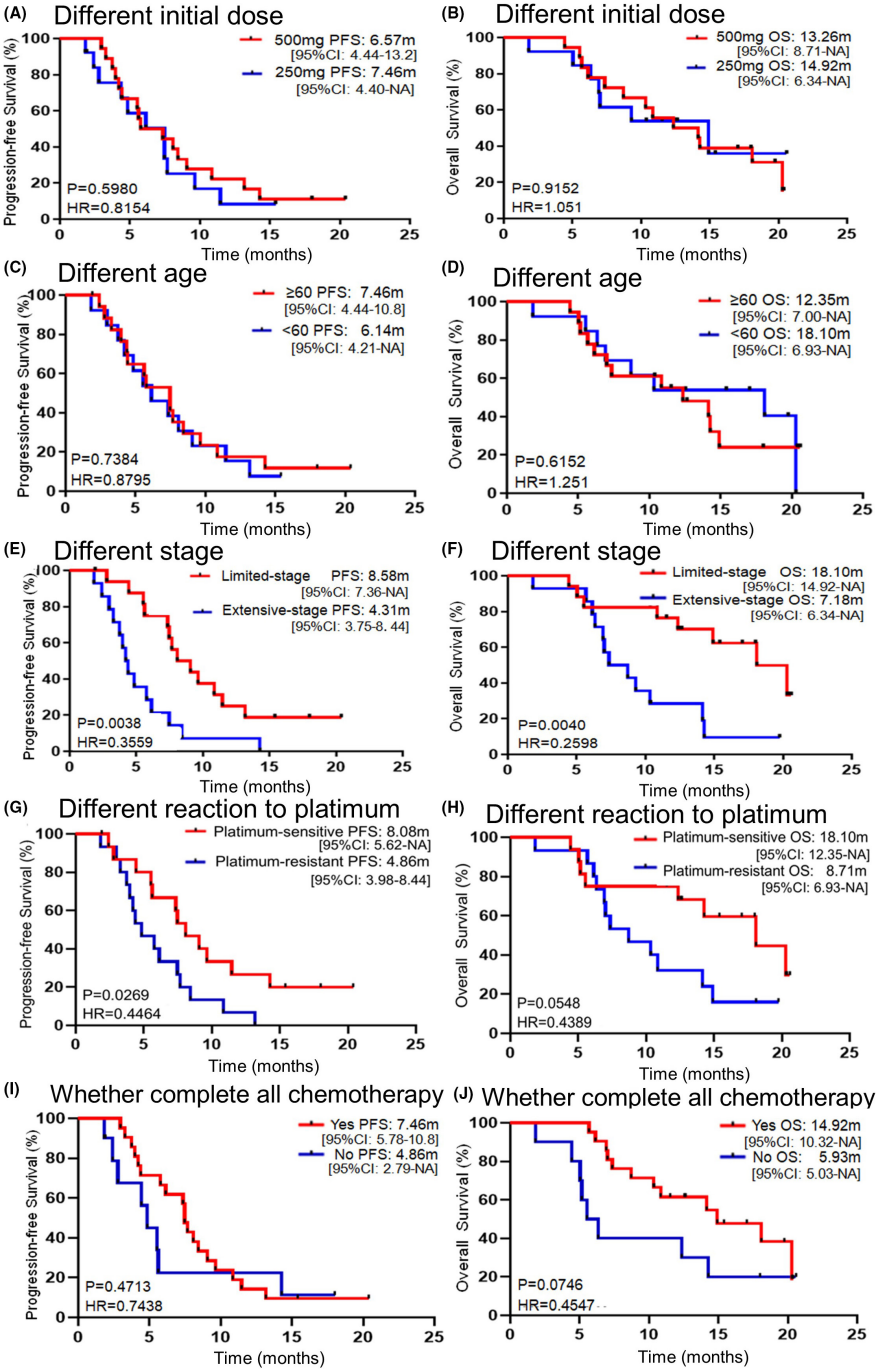
Subgroup analysis of PFS and OS of these enrolled patients. (A,B) Kaplan–Meier plots of PFS and OS in different initial dose. (C,D) Kaplan–Meier plots of PFS and OS in different age. (E,F) Kaplan–Meier plots of PFS and OS in different stage. (G,H) Kaplan–Meier plots of PFS and OS in different reaction to platimum. (I,J) Kaplan–Meier plots of PFS and OS in whether complete all chemotherapy.

The detailed treatment outcomes of the 31 patients were depicted in the swimmers plot (Figure.1A). As of September 1, 2020, 3 patients were still in the group, while 28 patients discontinued the treatment (1 was intolerant, 5 died during this study, and 22 had disease progression or continued to receive the subsequent treatment). Of the 31 patients, 2 were not included in tumor response evaluation, 1 due to death and the other due to intolerance. Among the remaining 29 patients who completed at least 2 cycles of treatment and were available for tumor response evaluation, none achieved complete response, while 8 patients achieved partial response and 20 patients were evaluated as stable disease, giving the ORR and DCR 27.59% (8/29) and 96.55% (28/29), respectively (Figure 1B). The median PFS of the total population was 7.36 months (95% confidence interval [CI]: 5.39–9.33) (Figure 1C), and the median OS was 14.16 months (95% CI: 9.52–18.80) (Figure 1D). In the subgroup analysis, initial dose of apatinib and age did not affect PFS and OS (Figure 2A–D). On the other hand, the patients with limited‐stage disease had a longer PFS than those with extensive‐stage disease (8.58 vs. 4.31 months, *p* = 0.0038) (Figure 2E) and OS (18.10 vs. 7.18 months, *p* = 0.0040) (Figure 2F). In platinum‐sensitive and platinum‐resistant subgroups, the median PFS was 8.08 vs. 4.86 months (*p* = 0.0269), and the median OS was 18.10 vs. 8.71 months, respectively (*p* = 0.0548) (Figure 2G, H). The DCR and ORR of the platinum‐sensitive group was 93.33% (14/15) and 33.33% (5/15), respectively, while those of the platinum‐resistant group were 100% (14/14) and 21.43% (3/14), respectively. Moreover, complete or incomplete four cycles of chemotherapy had no significant correlation with PFS (7.46 vs. 4.86 months, *p* = 0.4713) and OS (14.92 vs. 5.93 months, *p* = 0.0746) (Figure 2I, J).

### 3.3 Treatment duration of apatinib and OS

**FIGURE 3:**
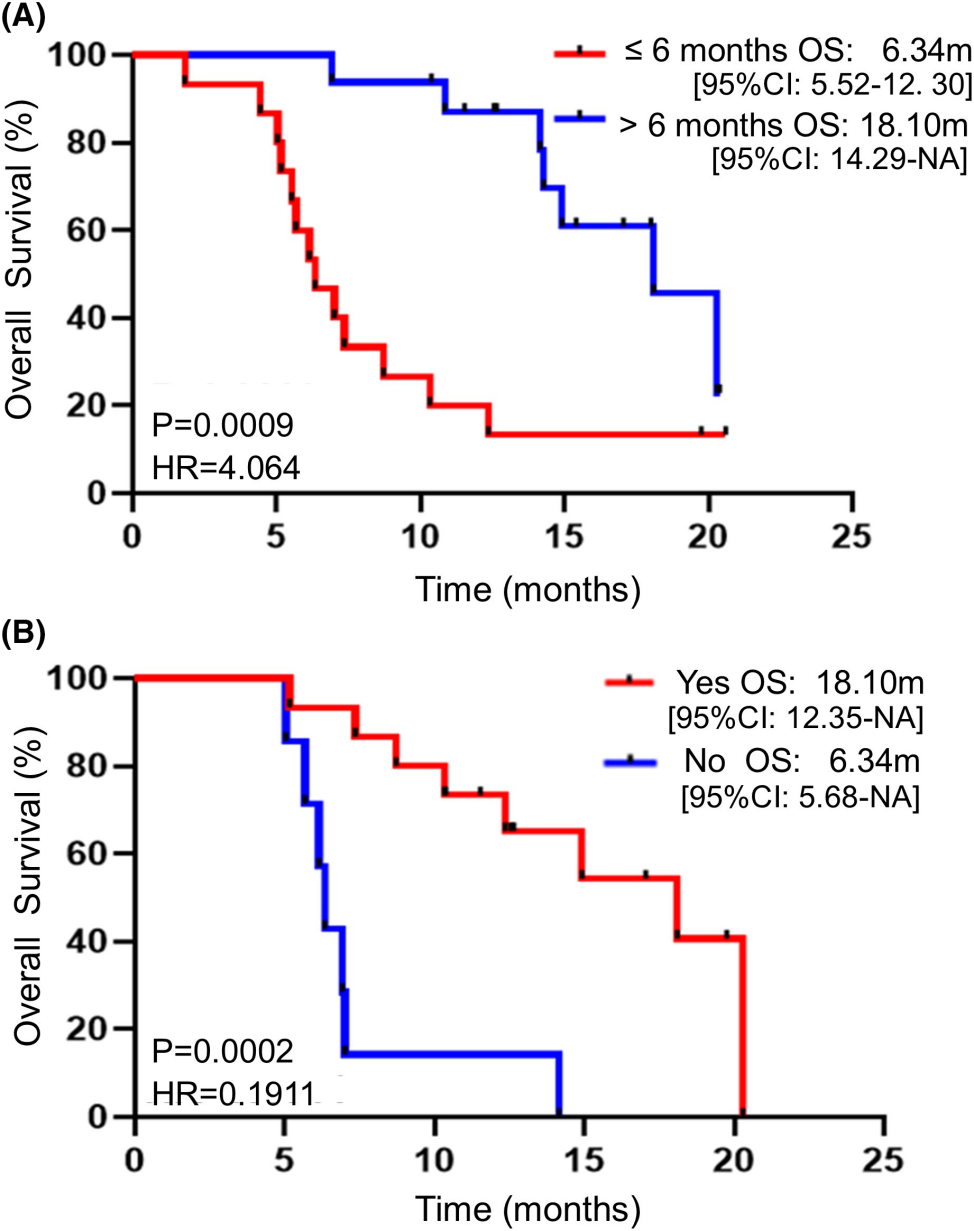
Subgroup analysis of the OS of patients with different treatment characteristics. (A) Survival of patients treated with apatinib for different durations. (B) Survival of patients receiving chemotherapy after disease progression.

In the current study, the median treatment duration of apatinib was 7.36 months, and the median duration of single apatinib maintenance therapy was 4.93 months. In the cohort, 15 patients used apatinib for less than 6 months, and 16 patients used it for more than 6 months. The difference in OS between the two groups of patients during the entire treatment process was statistically significant (6.34 vs. 18.10 months, *p* = 0.0009) (Figure 3A).

### 3.4 Subsequent therapy and OS

Next, 22/31 patients discontinued treatment due to disease progression. Among them, 15 patients accepted subsequent chemotherapy, and seven patients did not accept any subsequent anti‐cancer therapies. In this study, we observed that patients who accepted subsequent chemotherapy had longer OS than those who refused the regimen (18.10 vs. 6.34 months, *p* = 0.0002) (Figure 3B).

### 3.5 Multiple metastases and survival

Organ metastasis analyses revealed that 9/31 (29.03%) patients had brain metastasis, but no significant clinical symptoms, 4/21 (12.9%) patients had liver metastasis, 12/31 (38.71%) patients had lymph node metastasis, 5/31 (16.13%) had adrenal metastasis, and 2/31 (6.45%) had bone metastasis (Table S2). The stratified analysis of survival for different metastasis subgroups was performed in this study (Figure S2). Among all the organ metastases, bone metastasis had the worst prognosis (PFS 2.55 months, OS 4.59 months). However, only two patients had bone metastasis, thereby requiring additional clinical data for verification of this result. Significant differences were not found in the stratified analysis of survival for other metastasis subgroups.

### 3.6 Safety

The most common adverse events (AEs) were neutropenia (41.94%, 13/31), followed by leucopenia (35.48%, 11/31), and thrombocytopenia (25.81%, 8/31). The other common AEs were proteinuria (22.58%, 7/31), diarrhea (22.58%, 7/31), hypertension (22.58%, 7/31), and hand‐foot syndrome (22.58%, 7/31). Almost all the patients had different AEs, but most of the AEs were mild and tolerable. The incidence of grade 3–4 AEs was observed in 12 (38.71%) patients, of which neutropenia (19.35%, 6/31), leucopenia (9.68%, 3/31), and proteinuria (6.45%, 2/31) were most common. The apatinib‐related AEs, proteinuria, hypertension, and hand‐foot syndrome, were tolerable. No treatment‐related deaths were reported. The AEs are described in Table 2.

**TABLE 2:** Adverse events during the treatment phase in 31 patients

Adverse events	Any grade (*n*, %)	Grade 3–4 (*n*, %)
Hematologic indicators		
Leukopenia	11(35.48%)	3(9.68%)
Neutropenia	13(41.94%)	6(19.35%)
Thrombocytopenia	8(25.81%)	1(3.23%)
Anemia	4(12.90%)	0(0.00%)
Decreased hemoglobin	2(6.45%)	0(0.00%)
Liver function		
Elevated ALT or AST	5(16.13%)	0(0.00%)
Hypoproteinemia	6(19.35%)	0(0.00%)
Alkaline phosphatase	4(12.90%)	0(0.00%)
Elevated bilirubin	5(16.13%)	0(0.00%)
Renal function		
Proteinuria	7(22.58%)	2(6.45%)
Uric acid	1(3.23%)	0(0.00%)
Bloodlipid		
Hypertriglyceridemia	3(9.68%)	1(3.23%)
High/low density lipoprotein cholesterolemia	2(6.45%)	0(0.00%)
Gastrointestinal tract		
Reflux esophagitis	1(3.23%)	0(0.00%)
Gastritis	1(3.23%)	0(0.00%)
Diarrhea	7(22.58%)	0(0.00%)
Oral mucositis	3(9.68%)	0(0.00%)
Nausea, vomiting	6(19.35%)	0(0.00%)
Respiratory organs, chest and mediastinum		
Cough	2(6.45%)	2(6.45%)
Hemoptysis	1(3.23%)	0(0.00%)
Pneumonia	4(12.90%)	1(3.23%)
Pulmonary infection	1(3.23%)	1(3.23%)
Pericardial effusion	1(3.23%)	0(0.00%)
Cardiovascular		
Hypertension	7(22.58%)	1(3.23%)
Skin or epidermal tissue		
Hand‐foot syndrome	7(22.58%)	0(0.00%)
General conditions		
Fatigue	3(9.68%)	0(0.00%)
Anorexia	4(12.90%)	1(3.23%)

Abbreviations: ALT, alanine aminotransferase; AST, aspartate aminotransferase.

### 3.7 Dose adjustment of apatinib

Compared with the patients using 500 mg apatinib as the initial dose, the occurrence of treatment‐related AEs in the subgroup of patients using 250 mg initial apatinib was more optimistic. Consequently, 7/31 (22.58%) cases of intermittent medication, including four patients in the 500 mg dose group and three in the 250 mg dose group, were detected. Among the 18 patients (18/31) receiving an initial dose of apatinib 500 mg, 15 patients required dose reduction once (apatinib 250 mg/day) because of grade 3 or 4 toxicities, and two patients required second dose reduction to apatinib 250 mg every other day due to severe hypertension or neutropenia. The average duration for using apatinib 500 mg was 1.115 months. Among the 13 patients with an initial dose of apatinib 250 mg, only two patients accepted dose reduction to apatinib 250 mg every other day because of severe diarrhea (Table S3). Therefore, compared with apatinib dose of 500 mg/d, the dose of 250 mg/day might have better tolerance. Importantly, apatinib 250 mg had a promising efficacy in our study (Figure 1B), indicating that dose reduction of apatinib had no effect on apatinib.

## 4 DISCUSSION

Over the past two decades, antiangiogenic therapy has gained increasing attention because of its success in resisting multiple vascularized malignant tumors, such as colorectal cancer, NSCLC, and hepatocellular carcinoma.^21^, ^22^, ^23^ SCLC is one of the highly vascularized tumors with VEGF over expression in almost 80% of the tumor tissues.^5^ However, the role of antiangiogenic drugs in the treatment of SCLC is yet controversial. In 2009, the antiangiogenic agent thalidomide was reported to have no positive effect in improving the survival of advanced SCLC patients compared with the placebo group but increased the risk of thrombosis.^9^ In another large, randomized trial, sorafenib combined with platinum‐based chemotherapy showed a disappointing efficacy in treating extensive‐stage SCLC with significant toxicity.^24^ Interestingly, sunitinib, as maintenance therapy, prolonged the PFS (3.7 vs. 2.1 months) and OS (9 vs. 6.9 months) of extensive‐stage SCLC patients compared with placebo.^25^ Thus, additional clinical studies are needed to explore the role of antiangiogenic drugs in the treatment of SCLC.

Herein, we used an oral small molecular antiangiogenic TKI apatinib in combination with chemotherapy as second‐line therapy for the treatment of advanced pretreated SCLC. Regarding the potential mechanism of this combination therapy, firstly, since VEGF plays a key role in angiogenesis and apatinib could exert its antigenic effect by inhibiting its induced endothelial cell proliferation and migration by targeting VEGFR2 with high selectivity.^26^ And apatinib can also inhibit the growth of SCLC by affecting several pathway‐mediated mechanisms, such as PI3K/Akt and Ki‐67/CD31.^27^ In addition, one of the reasons for chemoresistance is that transport proteins on the cell membrane will expel chemotherapeutic drugs that enter the interior of tumor cells.^28^ Studies have shown that when apatinib is used in combination with chemotherapy drugs, it can significantly inhibit the efflux function of transporters, increase the sensitivity of drug‐resistant cells to drugs, even reverse the multidrug resistance of cells, and enhance the anti‐tumor efficacy of drugs.^29^ At present, apatinib has been used in combination with a variety of chemotherapeutic drugs and achieved good therapeutic effects in many tumors.^30^, ^31^ Therefore, we propose that the addition of apatinib to the second‐line chemotherapy of SCLC may improve the efficacy.

In our study, the majority of the patients presented partial response or stable disease. This new regimen showed better efficacy and tolerable toxicity for second‐line treatment of SCLC patients compared with the standard second‐line chemotherapies. In our study, PFS (7.36 months) and OS (14.16 months) were significantly longer than other therapies, such as topotecan, amrubicin, irinotecan, etc. Moreover, no intolerable AEs were observed in our study compared with other standard second‐line regimens (Table S1).^32^, ^33^, ^34^, ^35^, ^36^, ^37^, ^38^ Among these AEs, proteinuria, hypertension, and hand‐foot syndrome might be correlated with apatinib, which was consistent with that reported previously.^39^ Also, apatinib‐specific AEs were mild and tolerable.

A similar retrospective study investigated the efficacy of induction chemotherapy in combination with apatinib (250 mg/day) as maintenance therapy in extensive‐stage SCLC and found that the PFS was 8.3 months and the OS was 17 months.^40^ In another similar prospective study, apatinib (250 mg/day) was administered during the chemotherapy interval and as maintenance therapy after 4–6 cycles in treating extensive‐stage SCLC; the results indicated that the PFS was 7.8 months and the OS was 12.1 months.^12^ In our study, the subgroup analysis revealed that the PFS and OS of the extensive‐stage SCLC patients were 4.31 months and 8.4 months, respectively. These findings were inferior to those reported previously. This difference may be due to the reason that apatinib maintenance therapy was performed for patients with extensive‐stage SCLC who did not suffer from disease progression after first‐line treatment in the previous studies, whereas in our study, the patients received the treatment regimen after the failure of first‐line chemotherapy, rendering a poor physical state of the patients. Strikingly, the benefit of apatinib for SCLC is encouraging, as assessed by our subgroup analysis, wherein the OS of the patients receiving apatinib more than 6 months was significantly longer than those receiving apatinib for less than 6 months, although subsequent therapy was associated with the OS in our subgroup analysis. The correlation analysis between distant metastasis and survival revealed that only bone metastasis was significantly correlated with survival. In the study conducted by Yan et al., brain metastasis was demonstrated to be significantly correlated with survival, which was not observed in our study.^40^ Nonetheless, the sample size of our study and that of Yan et al. was small, which inevitably resulted in large deviation. Therefore, the association between distant metastasis and survival should be further explored via clinical data.

The recommended dose of apatinib for advanced gastric cancer is 850 mg/day.^41^ However, the dissolution and absorption of drugs in gastric cancer patients are affected by the specific diseases of the digestive tract, and hence, the dose of apatinib for gastric cancer cannot be used as the optimal treatment dose for lung cancer.^42^ Previous animal model studies have indicated that low‐dose apatinib increases the infiltration of tumor lymphocytes and alleviates hypoxia in vivo, which in turn inhibits tumor growth.^43^ The results of other studies on the dose of apatinib indicated that a low dose (250 mg/day) has similar efficacy and fewer AEs compared with high dose.^44^, ^45^ In the current study, 250 mg apatinib had efficacy similar to 500 mg, but better tolerance. Thus, our findings indicated that apatinib 250 mg could be a potential option as a safe dose to combine with chemotherapy for the treatment of advanced SCLC. However, it should be noted that the combination of apatinib and chemotherapy inevitably increases the incidence of AEs because chemotherapy may also lead to AEs, such as bone marrow suppression, abnormal liver function, and digestive system abnormality. For this new attempt, additional attention should be paid to treatment‐related AEs.

As a highly selective VEGFR2 inhibitor, apatinib shows multidrug resistance and is also resistance to angiogenesis. In addition, it acts on tumor cells directly and enhances the sensitivity of chemotherapeutics.^46^ These phenomena could be ascribed to the difference between apatinib and other antiangiogenic drugs in the treatment of SCLC and makes the combination of apatinib with chemotherapy a promising option for SCLC treatment. The current study further confirms the feasibility of this combination strategy. Therefore, in the future, randomized controlled trials with a large sample size would be conducted to develop an optimal combination regimen with better efficacy and less toxicity for this fatal disease. Furthermore, we analyzed biomarkers such as NSE and CEA in some of the enrolled patients. The results indicated that the NSE and CEA for the majority of patients showed decreased trend at the point of best tumor response and increased trend when the disease progressed (Figure S3), but this trend was not absolutely. This reflects the disease predictive value of NSE and CEA in SCLC to a certain extent, but this is only a small sample study, which still needs to be further verified with a larger sample size. In our study, more than half of patients has limited stage disease (17/31, 54%), indicating limited disease burden, but it could reflect the efficacy to a certain extent. According to the 8th edition of TNM staging, we divided the target lesions of limited‐stage patients into two groups of less than 3 cm and more than 3 cm to compare the clinical efficacy (Table S4). A total of 17 limited‐stage patients, one of them was out of the group due to intolerance, 16 patients can be used for efficacy evaluation. Among them, 11 cases with target lesions>3 cm were relatively large lesions, and five cases with lesions ≤3 cm were relatively small lesions. Based on the calculation results, there was no significant difference in PFS (8.08 m vs. 9.63 m, *p* = 0.77) and OS (18.10 m vs. 20.30 m, *p* = 0.93) between the two groups. The results showed that patients had limited stage disease with small disease burden does not have much impact on the efficacy evaluation, but more large‐scale research data are needed to confirm this point.

In addition, we estimated that the proportion of patients with this limited stage ranged from 20% to 40% by reviewing previous studies on second‐line treatment of SCLC.^37^, ^38^, ^47^, ^48^ The high proportion of 55% of our limited‐stage patients may be related to the small sample size and randomization. Furthermore, due to our randomization, the overall PFS was 7.36 m and the OS was 14.16 months. At the same time, in order to avoid the interaction between limited stage and extensive stage, we also conducted some subgroup analysis, the results showed that the PFS of limited stage was 8.58 m, OS was 18.1 m, and the PFS of extensive stage was 4.31 m, OS was 7.18 m. Our analysis was more detailed compared with other publications on SCLC patients with second‐line single‐agent chemotherapy,^32^, ^33^, ^34^, ^35^, ^36^, ^37^, ^38^ but it still needs more large‐scale research data to confirm this point.

Nevertheless, the present study has some limitations. In addition to the small sample size, this study did not have a control group. Thus, it could not be concluded that the combination regimen is superior to apatinib or chemotherapy alone in this study. However, the advantages of apatinib combined with chemotherapy have been proven in other lung cancer studies.^49^ Therefore, further prospective randomized investigations of the combination regimen within larger sample sizes are warranted.

## 5 CONCLUSION

Antiangiogenic therapy (apatinib) plus chemotherapy had encouraging efficacy. Also, no intolerable toxicity was observed for this new regimen, which should provide an insight into the current status of second‐line therapy in SCLC.

## AUTHORS’ CONTRIBUTIONS

Kewei Ma had full access to all of the data in the study and take responsibility for the integrity of the data and the accuracy of the data analysis. Conception and design: Kewei Ma and Yinghui Xu. Acquisition, analysis, or interpretation of data: Xu Wang, Chao Sun, Junya Song. Drafting of the manuscript: Yinghui Xu, Zhiru Gao. Statistical analysis: Hua He, Ye Guo, Shi Qiu, Xiaohui Ma. Critical revision of the manuscript for important intellectual content: All authors.

## FUNDING INFORMATION

K. Ma was supported by Wu Jieping Medical Foundation. Dr Y. Xu was supported by Youth Foundation of Norman Bethune Health Science Center of Jilin University (Grant ID: 2018B28) and Xisike Clinical Oncology Research Foundation (CSCO‐Haosen) (Grant ID: Y‐HS2017062).

## CONFLICT OF INTEREST

The authors declare that they have no conflicts of interest or competing interests.

## Supporting information


Figure S1
Figure. S2
Figure. S3
Table S1
Table S2
Table S3
Table S4

## Data Availability

Data are available upon request.
