# Supplementary material for: A phase II study of antiangiogenic therapy (Apatinib) plus chemotherapy as second‐line treatment in advanced small cell lung cancer
Source: Cancer Med. 2022 Sep 9;12(3):2979–89. doi: 10.1002/cam4.5217 (PMC9939110; doi:10.1002/cam4.5217)
Supplement: Supplementary file 1 — Figure S1 Figure. S2 Figure. S3 Table S1 Table S2 Table S3 Table S4 [file CAM4-12-2979-s001.docx]

**
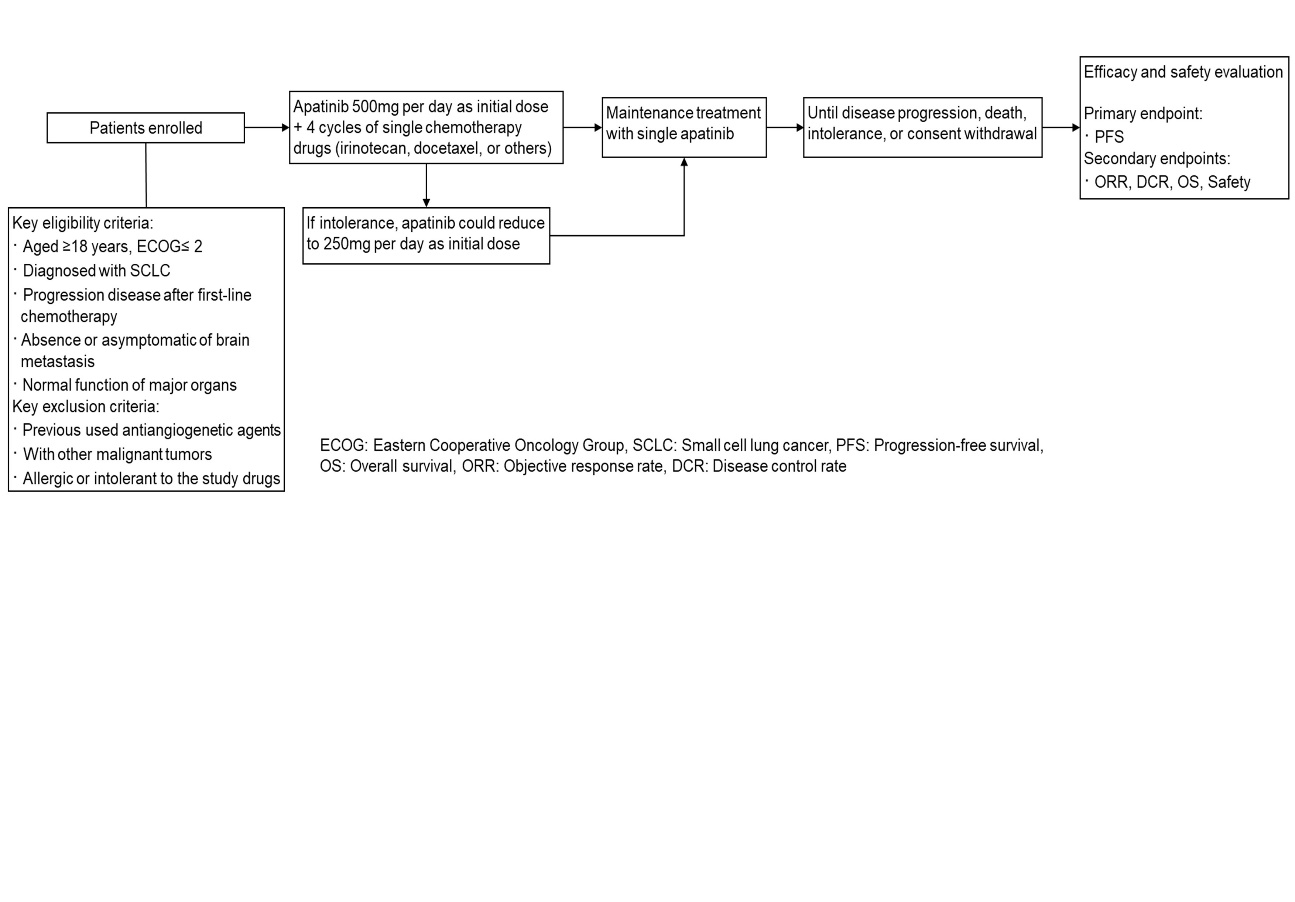
**

**Figure. S1.** Clinical study design


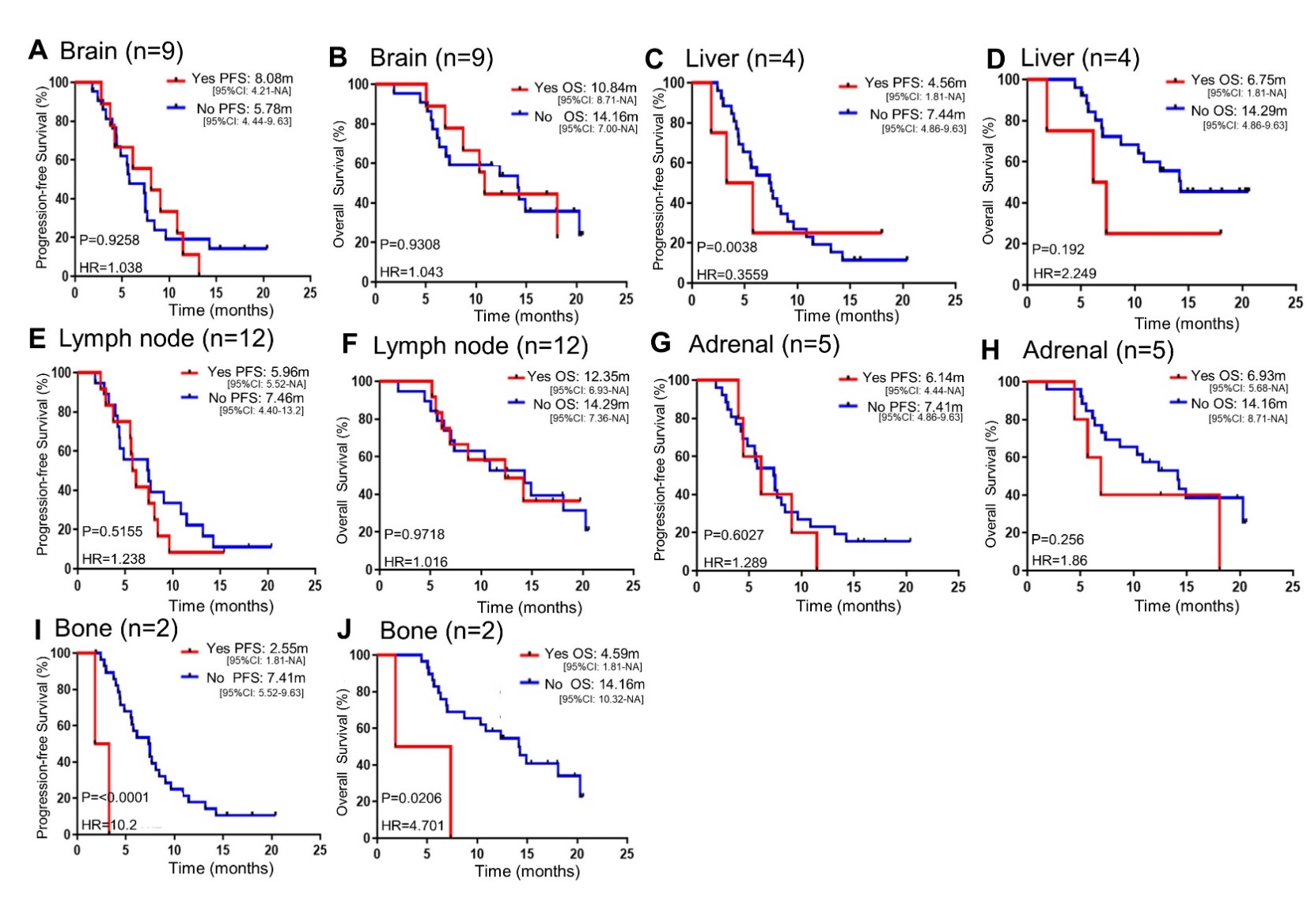


|  |  |
| --- | --- |

**Figure. S2.** Subgroup analysis of PFS and OS of different organ metastasis


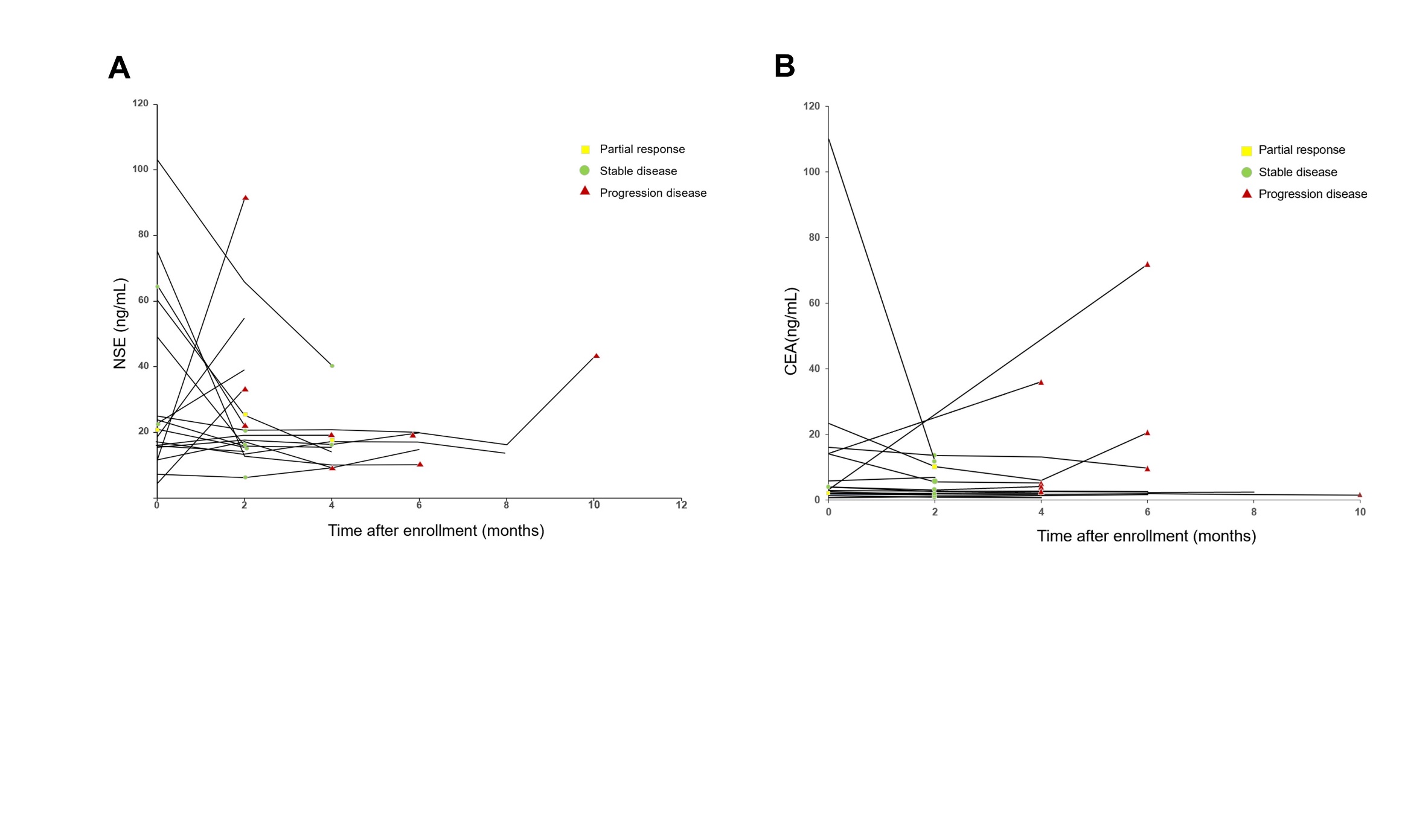


**Figure. S3.** Tumor marker changes in some enrolled patients (n=18). Tumor markers were not included in the monitoring of routine treatment at the time of study design, so tumor markers were not tested regularly for all patients.

| **Table S1.** Summary of the efficacy and safety of second-line single-agent treatment for SCLC | | | | | | | | | | | | | | | | | |
| --- | --- | --- | --- | --- | --- | --- | --- | --- | --- | --- | --- | --- | --- | --- | --- | --- | --- |
|  | | **Our study (N=31)** | | | | **Eckardt et al^32^ (N=151)** | | | | **Von Pawel et al^33^ (N=408)** | | | | **Morise et al^34^ (N=57)** | | | |
|  |  | **Apatinib + single chemotherapy** | | | | **Topotecan monotherapy** | | | | **Amrubicin monotherapy** | | | | **Irinotecan monotherapy** | | | |
| **Survival indicators** | |  | | | |  | | | |  | | | |  | | | |
| PFS (months) | | 7.36 | | | | 3.7 | | | | 4.1 | | | | 2.9 | | | |
| OS (months) | | 14.16 | | | | 8.8 | | | | 7.5 | | | | 5.3 | | | |
| **Adverse effects** | | All grade (%) | | Grade 3-4 (%) | | All grade (%) | | Grade 3-4 (%) | | All grade (%) | | Grade 3-4 (%) | | All grade (%) | | Grade 3-4 (%) | |
| Neutropenia | | 41.94 | | 19.35 | | - | | 87.8 | | 41.4 | | - | | 61.4 | | 36.8 | |
| Leucopenia | | 35.48 | | 9.68 | | - | | 75.3 | | 15.2 | | - | | 80.7 | | 33.3 | |
| Thrombocytopenia | | 25.81 | | 3.23 | | - | | 43.3 | | 21.1 | | - | | 21.1 | | 8.8 | |
| Anemia | | 12.90 | | 0 | | - | | 30.7 | | 15.9 | | - | | - | | - | |
| Nausea, vomiting | | 19.35 | | 0 | | - | | 2.7 | | - | | - | | 61.4 | | 8.8 | |
| Diarrhea | | 22.58 | | 0 | | - | | 2.7 | | - | | - | | 52.6 | | 7.0 | |
| Elevated ALT or AST | | 16.13 | | 0 | | - | | - | | - | | - | | 7.0 | | 0 | |
| Proteinuria | | 22.58 | | 6.45 | | - | | - | | - | | - | | - | | - | |
| Fatigue | | 9.68 | | 0 | | - | | 7.9 | | 10.5 | | - | | 31.6 | | 7.0 | |
| Anorexia | | 12.90 | | 3.23 | | - | | 2.7 | | - | | - | | - | | - | |
| Hand-foot syndrome | | 22.58 | | 0 | | - | | - | | - | | - | | - | | - | |
| Oral mucositis | | 9.68 | | 0 | | - | | - | | - | | - | | - | | - | |
| Hypertension | | 22.58 | | 3.23 | | - | | - | | - | | - | | - | | - | |
| **TABLE S1** (continued) Summary of the efficacy and safety of second-line single-agent treatment for SCLC | | | | | | | | | | | | | | | | | |
|  | | **Evans et al^35^ (N=89)** | | | | **Socinskin et al^36^ (N=116)** | | | | **Trigo et al^37^ (N=105)** | | | | **Zhao et al^38^ (N=34)** | | | |
|  |  | **Cabazitaxel monotherapy** | | | | **Pemetrexed monotherapy** | | | | **Lurbinectedin monotherapy** | | | | **Docetaxel monotherapy** | | | |
| **Survival indicators** | |  | | | |  | | | |  | | | |  | | | |
| PFS (months) | | 1.4 | | | | 1.2-1.5 | | | | 3.5 | | | | 1.7 | | | |
| OS (months) | | 5.2 | | | | 2.5-6.1 | | | | 9.3 | | | | 6.1 | | | |
| **Adverse effects** | | All grade (%) | | Grade 3-4 (%) | | All grade (%) | | Grade 3-5(%) | | All grade (%) | | Grade 3-4 (%) | | All grade (%) | | Grade 3-4 (%) | |
| Neutropenia | | 68.2 | | 56.8 | | - | | 24.8 | | 71.4 | | 45.7 | | - | | - | |
| Leucopenia | | 79.5 | | 52.3 | | - | | 18.2 | | 79.0 | | 28.6 | | - | | - | |
| Thrombocytopenia | | 59.1 | | 4.5 | | - | | 7.8 | | 43.8 | | 6.7 | | - | | - | |
| Anemia | | 94.3 | | 3.4 | | - | | 2.6 | | 95.2 | | 8.6 | | - | | - | |
| Nausea, vomiting | | 33.7 | | 3.3 | | - | | - | | 50.5 | | 0 | | - | | - | |
| Diarrhea | | 19.1 | | 2.2 | | - | | - | | 13.3 | | 0.9 | | - | | - | |
| Elevated ALT or AST | | - | | - | | - | | - | | - | | - | | - | | - | |
| Proteinuria | | - | | - | | - | | - | | - | | - | | - | | - | |
| Fatigue | | 29.2 | | 7.9 | | - | | 24.8 | | 58.1 | | 6.7 | | - | | - | |
| Anorexia | | - | | - | | - | | - | | - | | - | | - | | - | |
| Hand-foot syndrome | | - | | - | | - | | - | | - | | - | | - | | - | |
| Oral mucositis | | - | | - | | - | | - | | - | | - | | - | | - | |
| Hypertension | | - | | - | | - | | - | | - | | - | | - | | - | |

| **T****able S2.** Analysis of organ metastasis in total patients (n=31) | |
| --- | --- |
| **Organ** | **Number of patients (%)** |
| **Brain metastasis** |  |
| Yes | 9 (29.03) |
| No | 22 (70.97) |
| **Liver metastasis** |  |
| Yes | 4 (12.90) |
| No | 27 (87.10) |
| **Lymph node metastasis** |  |
| Yes | 12 (38.71) |
| No | 19 (61.29) |
| **Adrenal metastasis** |  |
| Yes | 5 (16.13) |
| No | 26 (83.87) |
| **Bone metastasis** |  |
| Yes | 2 (6.45) |
| No | 29 (93.55) |

| **Table S3.** Apatinib treatment process and proportion of patients | | |
| --- | --- | --- |
|  | **Number of patients** | **%** |
| **Drug interruption** | 7 | 22.58 |
| **Dose reduction** |  |  |
| **Initial dose 500mg (n=18)** |  |  |
| Reduction from 500mg to 250 mg per day | 15 | 83.33 |
| Reduction from 500 mg to 250 mg per day and then to every other day | 2 | 11.11 |
| **Initial dose 250mg (n=13)** |  |  |
| Reduction from 250mg per day to 250mg every other day | 2 | 15.38 |

| **Table S4.** The efficacy of target lesions in limited-stage patients into two groups (n=16) | | | | | | | |
| --- | --- | --- | --- | --- | --- | --- | --- |
|  |  | **n** | **ORR** | **Median(months)** | **0.95LCL** | **0.95UCL** | **P** |
| **mPFS** | **>3cm** | 11 | 45.45% | 8.08 | 5.62 | NA | 0.77 |
|  | **≤3cm** | 5 | 20.00% | 9.63 | 7.66 | NA |  |
| **mOS** | **>3cm** | 11 | 45.45% | 18.10 | 12.35 | NA | 0.93 |
|  | **≤3cm** | 5 | 20.00% | 20.30 | 14.92 | NA |  |
